# Prenatal intake of omega‐3 promotes Wnt/β‐catenin signaling pathway, and preserves integrity of the blood–brain barrier in preeclamptic rats

**DOI:** 10.14814/phy2.14925

**Published:** 2021-06-26

**Authors:** Asmaa M. ShamsEldeen, Marwa Nagi Mehesen, Basma Emad Aboulhoda, Laila Ahmed Rashed, Mohamed Mahmoud Elsebaie, Enas Ahmed Mohamed, Maha Mohammed Gamal

**Affiliations:** ^1^ Department of Physiology Faculty of Medicine Cairo University Cairo Egypt; ^2^ Department of Medical Pharmacology Faculty of Medicine Cairo University Cairo Egypt; ^3^ Department of Anatomy and Embryology Faculty of Medicine Cairo University Cairo Egypt; ^4^ Department of Biochemistry and Molecular Biology Faculty of Medicine Cairo University Cairo Egypt; ^5^ Department of Neurology Faculty of Medicine Cairo University Cairo Egypt

**Keywords:** blood–brain barrier, preeclampsia, ROS, sVEGFR‐1, Wnt/β‐catenin

## Abstract

**Background:**

Preeclampsia is a systemic, multi‐organ endotheliopathy, associated with oxidative injury to the blood–brain barrier (BBB). Preeclampsia initiates a cascade of events that include neuroinflammation. Recently, it was documented that Wnt/β‐catenin signaling pathway exerts neuroprotective effects and maintain BBB integrity. We investigate the protective effect of omega‐3 against neurovascular complication of preeclampsia and its relation to Wnt/β‐catenin signaling pathway.

**Methodology:**

After confirmation of day 0 pregnancy (G0), 24 adult pregnant female Wistar rats were divided into four groups control pregnant, pregnant supplemented with omega‐3, preeclampsia (PE); female rats received N (ω)‐nitro‐L‐arginine methyl ester (L‐NAME) (50 mg/kg/day SC from day 7 to day 16 of pregnancy for induction of preeclampsia) and PE rats supplemented with omega‐3. The intake of omega‐3 started on day zero (0) of pregnancy until the end of the study (144 mg/kg\day orally).

**Results:**

We found that omega‐3 supplementation significantly improved cognitive functions and EEG amplitude, decreased blood pressure, water contents of brain tissues, sFlt‐1, oxidative stress, proteinuria, and enhanced Wnt\β‐catenin proteins. Histological examination showed improved cerebral microangiopathy, increased expression of claudin‐1 and ‐3, CD31, and VEGF in the cerebral cortical microvasculature and choroid plexus in PE rats treated with omega‐3. A positive correlation between protein expression level of Wnt \β‐catenin and cognitive functions, and a negative correlation between claudin‐5 relative expression, claudin‐1 and ‐3 area % from one side and water content of the brain tissues from the other side were observed.

**Conclusion:**

Wnt/β‐catenin signaling pathway suspected to have an important role to improve BBB integrity. Neuroprotective, antioxidant, and anti‐inflammatory effects of omega‐3 were observed and can be suggested as protective supplementation for preeclampsia.

## Graphical abstract

Disruption of tight junction structure in BBB leads to brain edema and enhances vascular leakiness. Negative‐regulation of Wnt/β‐catenin signaling decreased its downstream claudin‐1 and ‐3 in preeclamptic rat model. Meanwhile, omega‐3 intake enhanced Wnt/β‐catenin signaling pathway that was associated with increased claudin‐1 and ‐3 expression, reducing Vasogenic edema together with improved cognitive function.

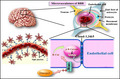

## 1 INTRODUCTION

Preeclampsia is the newly developed hypertension (140/90 mm Hg) and proteinuria (>300 mg/day) after 20 weeks of gestation in previously normotensive, nonproteinuric women (Lambert et al., 2014). Defective placentation and failure of trophoblast invasion could result in creating local placental ischemic hypoxic changes that drive systemic complications (Palei et al., 2013).

Disruption of the blood–brain barrier (BBB) and vasogenic brain edema are co‐existed complications of preeclampsia (Loureiro et al., 2003). The cerebral endothelial cells of the BBB are characterized by the presence of unique tight junctions (TJs), lack of fenestrations, and low permeability (Hammer & Cipolla, 2015).

Activation of β‐catenin in brain endothelial cells is essential for development and maintenance of BBB integrity (Tran, Zhang, et al., 2016). In addition, dysfunction of Wnt/β‐catenin signaling pathway in many diseases such as Alzheimer disease highlights the importance of this pathway in regulating the permeability of the BBB (Liu et al., 2014). Tran and colleagues (2016) reported that BBB breakdown and the increase in permeability occur secondary to defective expression and transcription activity of β‐catenin and the subsequently diminished claudin‐1 as well as claudin‐3 expression. Enhanced β‐catenin signaling in endothelial cells (ECs) of the choroid plexus in mice could induce autonomous expression of tight junction protein such as claudin‐5 (Wang et al., 2019). Claudin‐5 regulates and maintains integrity of brain microvasculature, it is responsible for “sealing” of TJs that contributes to their permeability (Ma et al., 2017). Deficiency of claudin‐5 increases permeability especially for small molecules (Yamazaki et al., 2016). On the contrary, disrupted endothelial β‐catenin downregulates the expression of claudin‐1 and −3 in adult brain endothelial cells (Khiem et al., 2016), thus indicating that maintained integrity of BBB depends on β‐catenin signaling.

Wnt/β‐catenin signaling pathway is inhibited and significantly decreased in placental tissues of preeclamptic patients compared to control group (Li, 2017). On the contrary, enhanced activation of Wnt/β‐catenin pathway contributes to trophoblast disorders such as choriocarcinoma (Zhang et al., 2017). However, the role of Wnt/β‐catenin in breakdown of BBB during preeclampsia needs further investigation.

In some clinical studies, the association between development of preeclampsia and low intake of omega‐3 long‐chain polyunsaturated fatty acids (LCPUFA) was observed. LCPUFA, particularly docosahexaenoic acid (DHA), 22:6 n‐3, stimulates the expression of pro‐angiogenic factors (Johnsen et al., 2011). Moreover, decreased LCPUFA level was associated with elevation of soluble fms‐like tyrosine kinase (sFlt‐1 or sVEGFR‐1 [soluble vascular endothelial growth factor receptor‐1]) levels in human preeclamptic females (Kulkarni et al., 2011). Hong et al., (2015) elucidated the neuroprotective effect of DHA against disruption of BBB in rats exposed to middle cerebral artery occlusion. Also, Zhang et al., (2017) demonstrated that dietary supplementation with omega‐3 can preserve and maintain the BBB integrity and TJs in neonates exposed to hypoxic/ischemic brain insult. However, the role of omega‐3 in protecting BBB integrity of preeclamptic mothers was not studied. The aim of this work was to investigate antioxidant and anti‐inflammatory effects of omega‐3 supplementation, and to evaluate its neuroprotective effect in maintenance of BBB integrity with its link to Wnt/β‐catenin signaling pathway during pregnancy, thereby modifying neurovascular complications of preeclampsia.

## 2 MATERIALS AND METHODS

### 2.1 Experimental animals and grouping

This study was carried out in the Physiology, Pharmacology, Biochemistry and Anatomy, and Embryology departments, Faculty of Medicine, Cairo University. The animals were purchased from and housed in the animal house of Research Institute of Ophthalmology (RIO).

Twenty‐four adult female Wistar rats approximately 8–10 weeks of age were included in this study. Animals were left for a few days to acclimatize to ordinary environmental living conditions in the animal house of RIO, as regards humidity, temperature, and dark ⁄ light cycles. They were kept in wire‐mesh cages (3 female rats in each) and had free access to food and water before starting the experimental procedure.

Fertile male Wistar rats were included for mating only following determination of estrus phase female reproductive cycle. The presence of spermatozoa in vaginal smears was considered as an indicator for the first day of gestation (G 0). Following copulation, female rats were housed singly. Experimental animal protocols and animal procedures complied with the highest International Criteria of Animal Experimentation in accordance to guidelines of Helsinki and were approved by animal house of Research Institute of Ophthalmology (RIO) for providing this work with animal and conducting the current study according to the Guide for the Care and Use of Laboratory Animals published by the US National Institutes of Health (NIH publication No. 85–23, revised 1996).

The pregnant female rats were randomly divided into the following four main groups in which each group included six rats: group I: control pregnant in which pregnant female rats were supplied with ad libitum access to food and water from the first day of gestation (G 0) to day 16 of pregnancy; group II: pregnant female rats supplied daily with omega‐3 (pregnant+Omega‐3). The daily recommended human dose is 1.4 g/day (Yessoufou et al., 2016); thus, it was converted intto rat dose of 144 mg/kg guided by Nair and Jacob study (2016) and was given to pregnant rats orally using gastric tube from the first day of gestation (G 0) to day 16 of pregnancy; group III: preeclampsia group (PE) in which pregnant female rats received N(ω)‐nitro‐L‐arginine methyl ester (L‐NAME) at a dose of 50 mg/kg/day subcutaneously from day 7 to day 16 of pregnancy for induction of preeclampsia (Motta et al., 2015); and group IV: PE+omega‐3 group in which the pregnant female rats received L‐NAME at a dose of 50 mg/kg/day subcutaneously from day 7 to day 16 of pregnancy for induction of preeclampsia **(**Motta et al., 2015 **)**, and they also supplied with omega‐3 orally from G 0 to day 16 of pregnancy using gastric tube at a dose of 144 mg/kg. At the day 16 of pregnancy and just before sacrifice, the female rats were subjected to assessment of cognitive function tests using open field, novel object tests and then estimation of blood pressure and electroencephalogram (EEG).

### 2.2 Calculating the dose of omega‐3

According to the Institute of Medicine Food and Nutritional Board, the recommended minimum adequate intake of omega‐3 PUFA for human pregnant female is 1.4 g/day (Yessoufou et al., 2016). In addition, guided by Nair and Jacob study (2016), the calculated rat dose was 144 mg/kg/day.

#### 2.2.1 Drug preparation and administration

1. Omega‐3; Omax3 (contains eicosapentaenoic acid [EPA] and DHA of 4:1 ratio) was purchased from Prevention Pharmaceuticals. 24 Arnett Avenue, Suite 107, Lambertville, NJ 08530. It was given orally using gastric tube.
2. L‐NAME hydrochloride (Sigma‐Aldrich, MO, USA) product number (483125‐M) powder was dissolved in saline solution and was given by subcutaneous injection.

#### 2.2.2 Chemicals

Ketamine HCl a product of Sigma‐Tec (Egypt) was used in the form of vials, each one contains 50 mg/ml.

### 2.3 All rats in all groups were subjected to assessment of cognitive function using

#### 2.3.1 Open field activity test for testing general locomotor activity, exploration habits, and anxiety

Open field apparatus consisted of a white square arena (that was 50 × 40) with walls 40 cm high (Lipták et al., 2013). The test sessions were recorded by a video camera placed just above the arena. On the day of the test, rats were transported to the testing room and left in their home cages for 1 h before the test. Each session started with placing the rat in the center of arena and allowing it to explore for 5 min. (Feyissa et al., 2017).

The apparatus was cleaned with 70% ethanol before testing each animal. During the test session we calculated number of lines crossed by the rat, duration taken by each rat to leave the central square, frequency of grooming and rearing (defined as standing on the hind limbs without touching the wall), and number of defecation boils.

#### 2.3.2 Novel object recognition test: reflects the use of learning and recognition memory

After acclimatization, rats were allowed to explore the arena freely for 10 min and they were monitored using a video camera. By the end of testing period, rats were returned to their cages for duration of 20 min. Then, the arena was cleaned with a wipe soaked with 70% ethanol to minimize olfactory cues before the next use. Two different objects were fixed near two corners of the arena, and then we started recording the video. Each female rat was allowed to investigate the arena and objects freely for 10 min. Then we stop recording the video. Each rat was placed back in their clean holding cages for a duration of 20 min. After using 70% ethanol for cleaning the arena and objects, trails were repeated with a novel object fixed instead of one of the familiar objects that were used before. Each rat was allowed to investigate the arena and objects freely for another 10 min. Finally we calculated the total investigation time of each rat of novel object test and of >50% means greater investigation of the novel object.

The percentage of total investigation time was calculated using the following equation (Denninger et al., 2018).

$$\mathrm{Percentage}\;\mathrm{of}\;\mathrm{total}\;\mathrm{investigation}\;\mathrm{time}=\;\frac{\mathrm{Time}\;\mathrm{with}\;\mathrm{novel}\;\mathrm{location}\;\mathrm{or}\;\mathrm{object}}{\mathrm{Time}\;\mathrm{with}\;\mathrm{novel}\;\mathrm{location}\;\mathrm{or}\;\mathrm{object}\;+\;\mathrm{Time}\;\mathrm{with}\;\mathrm{familiar}\;\mathrm{location}\;\mathrm{or}\;\mathrm{object}}\;x\;100$$

### 2.4 Measuring arterial blood pressure

Systolic blood pressures were measured to all rats. For better recording of blood pressure, the rat was prewarmed in an incubator for 15 min to increase the ambient temperature to 37℃ to maintain an adequate circulation in the rat tail vessels to measure systolic blood pressure reliably.

Blood pressure in rats was measured by LE 5001 pressure meter LSiLetica, scientific industries. This system is an electronic version of the traditional sphygmomanometer cuff method, used to determine human blood pressure indirectly.

### 2.5 Recording electroencephalogram (EEG)

At day 16 of gestation, after giving anesthesia (ketamine at a dose of 100 mg/kg i.p), each pregnant female rat was subjected to electroencephalogram (EEG) recording which was done guided by AD Instruments, 2012. EEG electrodes were placed under the scalp of the rat, one positive and the other negative on both sides of the skull with the third reference electrode at the back of the head corresponding to the base of the skull. Shielded, low‐weight, flexible cables, connecting the electrodes to the input dual bioamplifier (ml408; dbs337) was attached to examined rat. EEG was recorded on signal channel (channel1:source channel) with low‐frequency filter (LFF) of 0.1 Hz and high‐frequency filter (HFF) of 60 Hz with sampling rate of 1 KHz (Liu et al., 2016).

EEG was digitized and stored with the use of standard PC‐based hardware (ADInstrument). Power Lab v.7.3.7 was used to illustrate the recording diagram. EEG was subjected to offline analysis of average cyclic amplitude in mV of the source wave.

## 3 SAMPLES COLLECTION AND SCARIFICATION

At the end of the study period and 24 h before sacrifice, female rats were subjected for collection of 24‐h urine for measuring protein level. Just before scarification, blood samples were withdrawn from rat tail veins. Blood samples were collected; plasma was separated by centrifugation and then used for estimating levels of triglycerides, low‐density lipoproteins (LDL), cholesterol, high‐density lipoproteins (HDL), serum fms‐like tyrosine kinase(sFlt‐1/sVEGFR‐1) and reactive oxygen species (ROS), and serum tumor necrosis factor alpha (TNF‐α).

Then, animals were sacrificed by cervical dislocation, the skull was opened, and the brain was dissected into two cerebral hemispheres.

### 3.1 Measuring brain water content

Half of the brain samples were used for estimation of water content (6 in each group). Water content was quantitated by the wet‐weight/dry‐weight method. One brain hemisphere was gently blotted with tissue paper to remove the small quantities of adsorbent cerebrospinal fluid. The tissue samples were rapidly weighed with a basic precision scale (g), and then dried to constant weight in a vacuum oven at 105 C for 24 h to obtain the dry weight (Shigemori et al., 2006) then brain water content was estimated by the following equations (keep et al., 2012):

Watercontent=(wetweight‐dryweight)/dryweight

Then, the other half of brain hemispheres were used for estimating protein expression levels of brain endothelial nitric oxide (eNOS), inducible nitric oxide (iNOS), and matrix metalloproteinase‐9 (MMP‐9) (92‐kDa gelatinase B) by western blot analysis and Wnt/β‐catenin pathway by real‐time PCR. Part of brain samples were fixed in 10% formol saline for 24 h. Paraffin blocks were prepared for further investigation using hematoxylin and eosin (H&E) stain, immunohistochemistry for VEGF‐1 and CD31 for detection of angiogenesis, claudin‐1 and claudin‐3 for detecting the integrity of the BBB.

### 3.2 Measurement of total plasma cholesterol, high‐density lipoprotein cholesterol, low‐lipoprotein cholesterol

Total plasma cholesterol, high‐density lipoprotein cholesterol (HDL‐C), and low‐lipoprotein cholesterol (LDL) were measured using a quantitative enzymatic‐colorimetric test according to Shamseldeen et al. (2019).

### 3.3 Measurement of plasma triglycerides

Plasma triglyceride levels were assayed using triglyceride quantification kit according to the method adopted by Wahlefeld (1974).

### 3.4 Determination of serum FMS‐like tyrosine kinase (sFlt‐1)

sFlt‐1 was assayed using quantitative sandwich enzyme immunoassay technique using rat‐soluble Fms‐like tyrosine kinase receptor 1 ELISA Kit, Cat. MBS725733. Antibody specific for sFlt‐1 had been precoated onto a microplate. After pipetting standards and samples into the wells, a biotin‐conjugated antibody specific for sFlt‐1 was added to the wells. After washing, avidin‐conjugated horseradish peroxidase (HRP) was added to the wells. Awash was done to remove any unbound avidin‐enzyme reagent then a substrate solution was added to the wells and color develops in proportion to the amount of sFlt‐1/sVEGFR‐1 bound in the initial step. The color development was stopped and the intensity of the color was measured.

### 3.5 Determination of reactive oxygen species levels

ROS levels were determined using a quantitative sandwich ELISA kit (hereafter termed "analyte") in undiluted original rat serum.

### 3.6 Estimating serum TNF‐α level

The maternal serum TNF‐α level was determined using ELISA kits (R&D Systems, Minneapolis, MN) and the steps was done according to the manufacturer's instructions; then the data were analyzed using a Bio‐Plex™ system (Luminex Bio‐Plex™ 200 System, Bio‐Rad).

### 3.7 Measurement of urinary albumin level

Twenty‐four hours urine samples were collected using specialized cages (metabolic cages) to measure urinary albumin. Before this stage, rats were fed once before being deprived from food for 1 day, yet, still having free access to water. Assessment of urinary albumin was done by using Albuwell M Kit: Murine Microalbuminuria ELISA (DiaComp, USA).

### 3.8 Determination of ENOS, INOS, and MMP‐9 protein and WNT/β‐catenin levels using western blot technique

Proteins from brain tissues were extracted using a protein extraction kit. Proteins were extracted by using lysis buffer (radioimmunoprecipitation assay (RIPA) buffer with protease and phosphatase inhibitor) on ice for 30 min in a shaker as it was previously done in Aboulhoda et al., (2021), After centrifugation of the tissues at 16,000 *g* for 30 min at 4℃, the debris was removed. Then, the supernatants were transferred to other tubes for concentration determination and protein analysis. A concentration of nearly 20‐μg protein from each sample was loaded with an equal volume of Laemmli sample buffer. For ensuring protein denaturation, an aliquot of 7.5 μg proteins from each sample was boiled with Laemmli buffer at 95℃ for 5 min. Then, after loading samples into lanes in sodium dodecyl sulfate–polyacrylamide gel electrophoresis (SDS–PAGE), they were transferred to polyvinylidene (PVDF) membranes. The membranes were blocked with a formed composition of tris‐buffered saline with Tween 20 (TBST) buffer and 3% bovine serum albumin (BSA) for 1 h at room temperature and then incubated with primary antibodies (for iNOS, eNOS, MMP9 and Wnt/β‐catenin), 1:2000, Thermofisher, USA) overnight at 4℃. These antibodies were diluted in TBST. The formed blots were rinsed three to five times for 5 min with TBST. After that, the incubation with the peroxidase‐conjugated secondary antibody (Novus Biologicals) solution was done for 1 h at room temperature. The blots were rinsed three to five times for 5 min with TBST. Using an enhanced chemiluminescence (ECL) system (Clarity™ Western ECL substrate—BIO‐RAD, USA), the formed bands were visualized, their chemiluminescent signals were captured, and finally the targeted band intensity were analyzed using a ChemiDoc MP imager (BIO‐RAD, USA) and read against the control sample by normalization to the house keeping gene β‐actin.

### 3.9 Preparation of the brain capillary fraction and determination of claudin‐5 using quantitative real‐time PCR

After excision and homogenization in phosphate‐buffered saline (PBS), the rat brain homogenate was added to 32% dextran and centrifuged. The formed pellets by centrifugation were washed in PBS to give the capillary‐rich fraction (Boado & Pardridge, 1991). The latter was allowed to pass through an 85‐µm nylon mesh filter, and then applied to a column of glass beads (350–500 µm) that was washed with PBS. Finally, the brain capillaries attached to the glass beads were isolated by gentle shaking.

As described previously, total RNA was prepared from the brain capillary fraction using TRIzol reagent (Invitrogen, Carlsbad, CA), all procedures were done according to the manufacturer's instructions. Real‐time RT‐PCR was carried out with reverse‐transcribed cDNA that was formed from total RNA, a 2× SYBR Green PCR master mix (PE‐Applied Biosystems) and specific primers for claudin‐5 (NM_031701) sense TTAAGGCACGGGTGGCACTCACG and antisense TTAGACGTAGTTCTTCTTGTCG.

### 3.10 Histological examination

The brain tissue was kept in 10% formalin solution and subjected to hematoxylin and eosin (H&E) staining after routine paraffin block and histological preparation.

### 3.11 Immunohistochemical evaluation

Paraffin sections were cut into 5 μm thickness on poly‐l‐lysine‐coated slides. Brain sections were deparaffinized in xylene, rehydrated in descending grades of alcohol, and then incubated in hydrogen peroxide for blocking of endogenous peroxidase activity. The sections were then placed in citrate buffer in a microwave for antigen retrieval. In order to prevent nonspecific background staining, the sections were incubated in 1% BSA dissolved in PBS. This was followed by incubation with rabbit polyclonal anti‐Claudin 1 antibody (ab15098), anti‐Claudin 3 antibody (ab15102), anti‐VEGFA antibody (ab51745), Anti‐CD31 antibody (ab124432) (1:100, IHC‐P, Abcam®, Cambridge, MA, USA). The goat anti‐rabbit IgG (ab205718) secondary antibody was then applied. Diaminobenzidine tetrahydrochloride solution was then applied. The sections were then washed in distilled water and counterstained with Mayer's hematoxylin, followed by washing in tap water, dehydration, clearing, and mounting by DPX. Paraffin‐embedded mouse heart tissue was used as a positive control. Negative controls were processed via omission of the primary antibodies in the automated staining protocol. Morphometric analysis for the count of CD31 immuno‐positive capillaries and the area percentage of claudin‐1, claudin‐3, and VEGF immunohistochemical expression was performed in 6 nonoverlapping fields under magnification 400 in the standard measuring frame of 85,550 mm using Image J computer analysis system (J Image Pro Plus6.0, Media Cybernetics, Silver Spring, MD, USA).

### 3.12 Statistical methods

Data were coded and entered using the statistical package SPSS version 25. Data were summarized using mean and standard deviation. Comparisons between groups were done using unpaired *t* test or analysis of variance with multiple comparisons post hoc test in normally distributed quantitative variables while nonparametric Kruskal–Wallis test and Mann–Whitney test were used for non‐normally distributed quantitative variables. Correlations between quantitative variables were done using Spearman correlation coefficient. *p*‐values < 0.05 were considered as statistically significant.

## 4 RESULTS

### 4.1 Improved cognitive functions of both normal pregnant and preeclamptic female rats with the intake of omega‐3

While observing changes in estimated parameters from open field test, the decrease in number of line crossing, frequency of rearing, and percentage of total investigation time was significant in preeclampsia rats compared to control pregnant rats (Table 1).

**TABLE 1:** Omega‐3 supplementation improved cognitive functions of drug‐induced preeclamptic rats

	Control pregnant	Pregnant‐Omega−3	PE	PE‐Omega−3
No of line crossing	42.83 ± 4.96	51.5 ± 4.23	25.83 ± 5.42^a^, ^b^	44.17 ± 8.08^c^
Duration to leave central square (sec)	1.83 ± 0.75	1.83 ± 0.98	6.33 ± 1.37^a^, ^b^	3.83 ± 0.98^a^, ^b^, ^c^
Frequency of rearing	8.5 ± 1.87	8.33 ± 1.86	2.67 ± 0.82^a^, ^b^	5 ± 2.52^a^, ^b^, ^c^
Frequency of grooming	2.67 ± 1.75	3.33 ± 1.63	7.17 ± 1.17^a^, ^b^	6 ± 1.09^a^, ^b^
No of defecation boil	1.5 ± 1.38	3 ± 0.63	5.5 ± 1.05^a^, ^b^	4 ± 0.89^a^
Total investigation time of novel object test (%)	86.5 ± 11.02	87.17 ± 5.04	24.67 ± 5.47^a^, ^b^	53 ± 7^a^, ^b^, ^c^

Data show no of line crossing, frequency of grooming and rearing, no of defecation boils obtained from open field test and % of investigation time obtained from novel object test. Data are presented as mean ± SD.

^a^ Statistically significant compared to corresponding value in the control group (*p* < 0.05).

^b^ Statistically significant compared to corresponding value in the pregnant‐omega group (*p* < 0.05).

^c^ Statistically significant compared to corresponding value in the PE group (*p* < 0.05).

There was also a significant increase in duration taken to leave the central square, frequency of grooming and number of defecation boils in preeclampsia rats compared with control pregnant rats. In addition, number of line crossing, and percentage of total investigation time in preeclampsia pregnant rats taking omega‐3 were significantly increased, although there was increase in mean values of rearing in PE‐omega‐3 group this increase wasn't significant when compared to PE group.

A significant decrease in duration taken to leave Central Square was observed in PE‐omega‐3 compared to PE rats. Although frequency of grooming and number of defecation boils in PE‐omega‐3 were decreased, yet it showed a nonsignificant decrease compared to PE rats (Table 1).

### 4.2 Diverse health‐promoting actions of omega‐3 fatty acids involve blood pressure lowering effect

Mean systolic arterial blood pressure (SBP; mm Hg) showed no significant changes in normal pregnant rats taking omega‐3 (124.33 ± 11.47) compared to control normal pregnant rats (125.83 ± 9.46). SBP was significantly increased in PE rats (236.33 ± 19.33) compared to control normal pregnant rats (125.83 ± 9.46).

In addition, the intake of omega‐3 significantly decreased the mean values of SBP in PE‐omega‐3 (186 ± 5.12) compared to preeclampsia pregnant rats (236.33 ± 19.33).

### 4.3 Decreased amplitude of EEG waves secondary to intake of omega‐3

**FIGURE 1:**
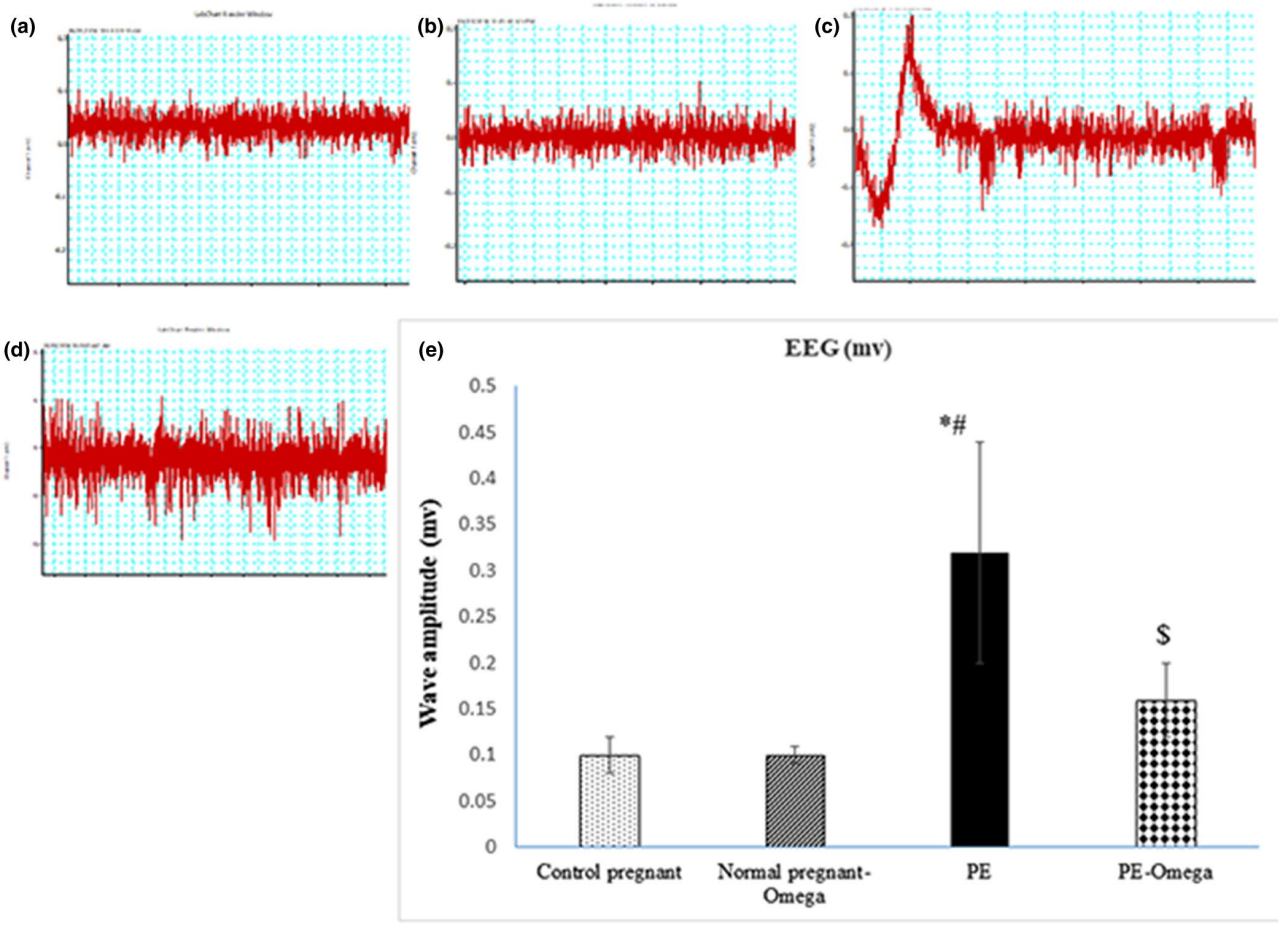
(a, b, c, d) the amplitude of EEG waves in all the studied groups, (a) for control pregnant, (b) for normal pregnant‐omega, (c) for PE, and (d) for PE‐omega. (e) Statistical analysis of the amplitude of EEG waves measured in mv. Data are presented as mean ± SD (*n* = 6),*: statistically significant relative to the control group (*p* < 0.05), #: statistically significant relative to the normal pregnant‐omega group (*p* < 0.05), $: statistically significant relative to the PE group (*p* < 0.05)

EEG amplitude measured in gestational day 16 showed no significant changes in normal pregnant rats compared to normal pregnant rats taking omega‐3. However, the amplitude was significantly increased in PE rats (0.32 ± 0.12) compared to control pregnant rats (0.1 ± 0.02). Intake of omega‐3 resulted in significant decrease in EEG amplitude in PE‐omega‐3 (0.15 ± 0.05) compared to PE rats (0.32 ± 0.12) (Figure 1).

### 4.4 Decreased water contents of brain tissues secondary to the intake of omega‐3:

**FIGURE 2:**
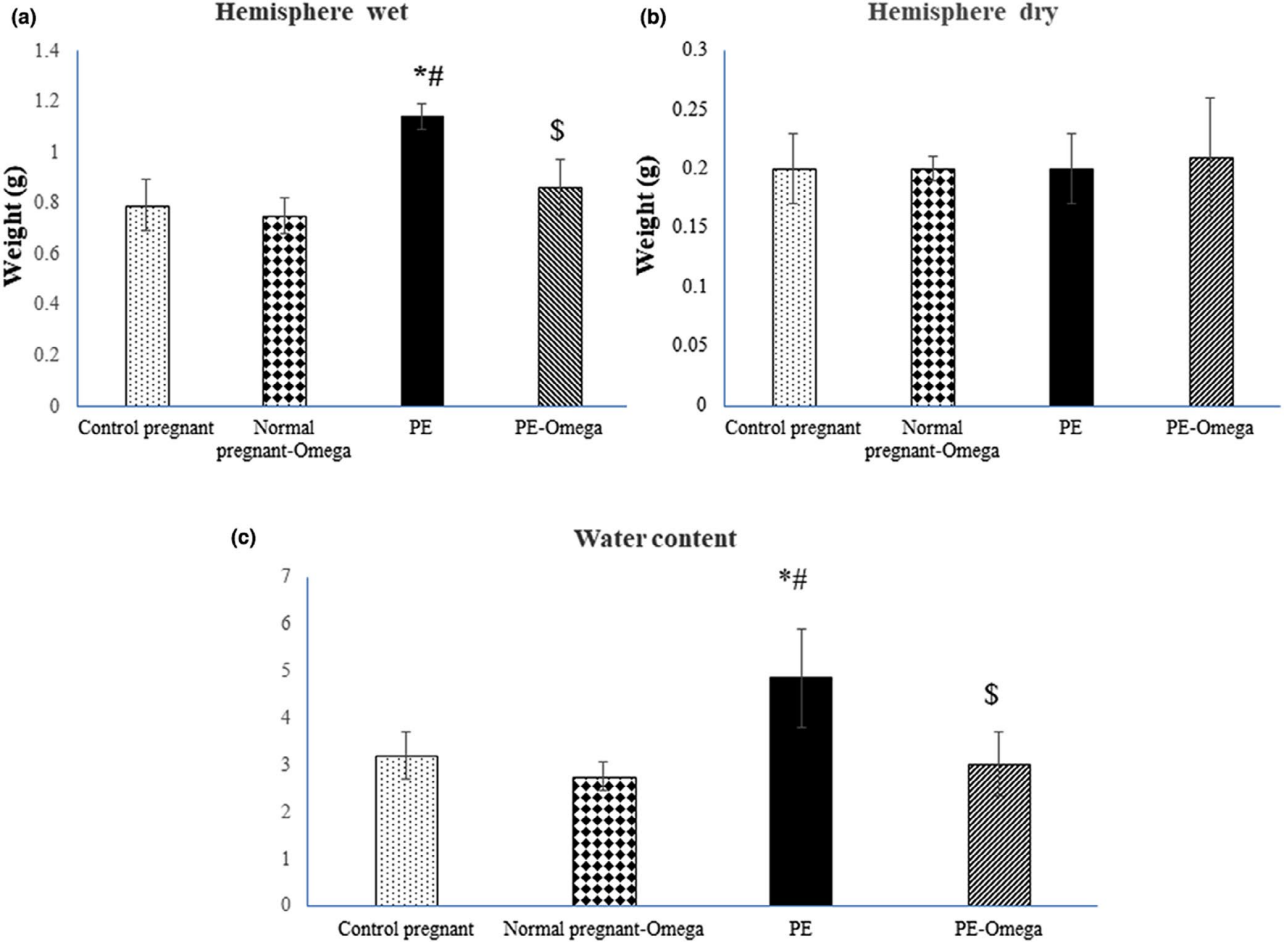
(a, b) The wet and dry brain weight (gm) and (c) presents the water contents in all the groups. Data are presented as mean ± SD (*n* = 6), *: statistically significant relative to the control group (*p* < 0.05), #: statistically significant relative to the normal pregnant‐omega group (*p* < 0.05), $: statistically significant relative to the PE group (*p* < 0.05)

Brain wet and dry hemisphere weights (g) showed no significant changes in normal pregnant rats compared to normal pregnant rats taking omega‐3. The results showed that brain wet hemisphere weight were significantly increased in PE rats (1.14 ± 0.09) compared to control pregnant rats (0.79 ± 0.1). The same parameter was significantly decreased in PE‐omega‐3 rats (0.86 ± 0.12) compared to PE rats (1.14 ± 0.09). After estimation of dry hemisphere weight, there was nonsignificant change observed between all the studied groups (Figure 2).

Water content in PE rats was increased (4.86 ± 1.05) compared with control pregnant rats (3.22 ± 0.50). In addition water content in PE‐omega‐3 rats (3.02 ± 0.68) was significantly decreased compared to PE rats (Figure 2).

### 4.5 Improved lipid profile in groups supplemented with omega‐3

HDL levels were increased and cholesterol, triglycerides, and LDL levels were decreased in normal pregnant rats taking omega‐3. But the increased HDL, the decreased cholesterol levels as well as triglycerides levels were not significant compared to control pregnant rats. However, the decrease in LDL was significant in normal pregnant rats taking omega‐3 compared to control pregnant rats.

Lipid profile showed significant decrease in HDL levels in PE rats compared to control pregnant rats, and the intake of omega‐3 significantly increased HDL level PE‐omega‐3 compared to PE group.

Cholesterol, triglycerides, and LDL levels were significantly increased in PE rats compared to control pregnant rats. However, in PE‐omega‐3, these parameters were significantly decreased compared to PE group (Table 2).

**TABLE 2:** Improved lipid profile, amelioration of sFlt‐1, ROS, TNF‐α, and proteinuria secondary to omega‐3 supplementation

	Control pregnant	Normal pregnant‐Omega	PE	PE‐omega−3
HDL (mg/dl)	43.67 ± 11.2	51.17 ± 2.93	23 ± 3.29^a^, ^b^	41 ± 4.56^c^
Cholesterol (mg/dl)	182.33 ± 8.87	149.67 ± 4.13	272.33 ± 53.18^a^, ^b^	182.33 ± 15.14^c^
Triglycerides (mg/dl)	87.5 ± 16.33	81.83 ± 6.43	149.5 ± 5.89^a^, ^b^	126.5 ± 18.56^a^, ^b^, ^c^
LDL (mg/dl)	128.17 ± 8.01	79.17 ± 9.13^a^	229.5 ± 38.39^a^, ^b^	125.5 ± 7.66^b^, ^c^
sFlt−1/s (ng/ml)	3.38 ± 1.15	3.06 ± 0.24	8.55 ± 1.23^a^, ^b^	3.9 ± 0.24^c^
ROS levels (nmol/l)	16.85 ± 2.88	9.25 ± 1.34	41.1 ± 10.96^a^, ^b^	20.7 ± 4.15^b^, ^c^
TNF‐α (pg/ml)	318.33 ± 19.91	291.33 ± 10.39	570.33 ± 22.62^a^, ^b^	422.5 ± 6.8^a^, ^b^, ^c^
24‐hour urinary protein (mg/ml)	6.08 ± 2.55	3.08 ± 0.47	33.33 ± 6.43^a^, ^b^	11.93 ± 1.43^a^, ^b^, ^c^

Data are presented as mean ± SD.

^a^ Statistically significant compared to corresponding value in the control group (*p* < 0.05).

^b^ Statistically significant compared to corresponding value in the normal pregnant‐omega group (*p* < 0.05).

^c^ Statistically significant compared to corresponding value in the PE group (*p* < 0.05).

### 4.6 Omega‐3 supplementation ameliorated sflt‐1, oxidative stress, and proteinuria

Intake of omega‐3 yielded a decrease in sFlt‐1 and TNF‐α level in control pregnant‐omega‐3 compared to control pregnant rats but this decrease was not significant. The results showed significant increase in levels of sFlt‐1 and TNF‐α in PE group compared to control pregnant rats and significant decrease in PE‐omega‐3 compared to PE rats. Similarly, ROS levels were significantly increased in PE rats compared to control pregnant rats and significantly decreased in PE‐omega‐3 compared to PE rats (Table 2).

Estimated 24‐h urinary protein levels were significantly increased in PE rats compared to control pregnant rats. The state proteinuria accompanied preeclampsia was not totally corrected with the intake omega‐3; however, it was significantly decreased in PE‐omega‐3 compared to PE rats (Table 2).

### 4.7 Omega‐3‐suppressed iNOS and MMP9 and enhanced eNOS expression in brain tissues of preeclamptic rats

Data depicted significant decreased of eNOS at the tissue levels in PE rats compared to control pregnant rats, while iNOS levels was significantly increased in PE rats compared to control pregnant rats. eNOS showed a significant increase in its value in PE‐omega‐3 compared to PE rats. In contrast to eNOS, iNOS levels were significantly decreased in PE‐omega‐3 compared to PE rats.

**FIGURE 3:**
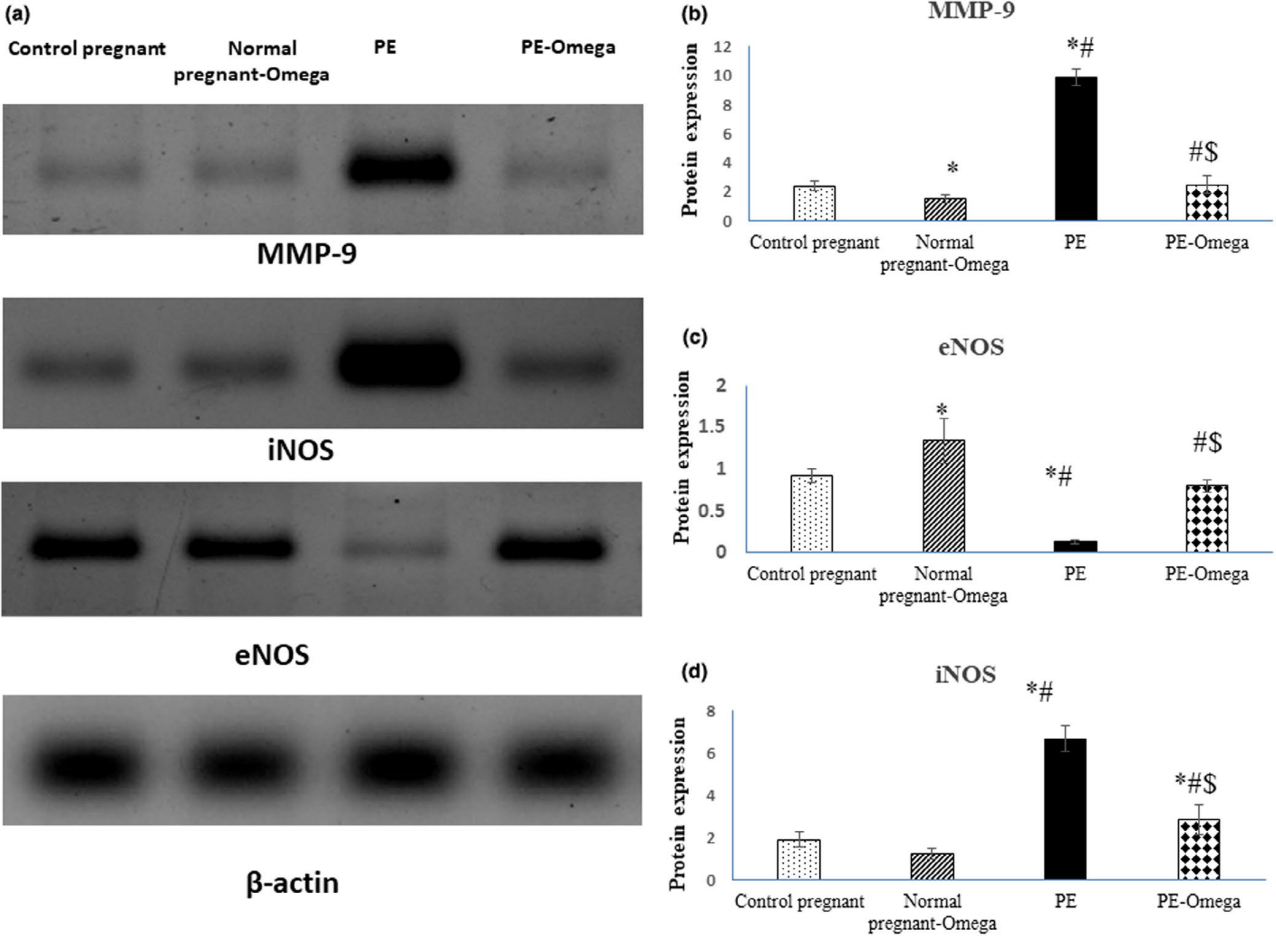
(a) Protein bands and (b, c, d) statistical analysis of MMP‐9, iNOS, and eNOS in relation to the house keeping gene β‐actin. Data are presented as mean ± SD (*n* = 6), *: statistically significant relative to the control group (*p* < 0.05), #: statistically significant relative to the normal pregnant‐omega group (*p* < 0.05), $: statistically significant relative to the PE group (*p* < 0.05)

MMP‐9 regulates degradation of tight junction protein and the extracellular matrix. Western blot analysis of MMP‐9 level was measured in all the groups. Data showed significant increase of MMP‐9 in PE rats compared to control pregnant rats and significant decreased in PE‐omega‐3 compared to preeclampsia pregnant rats (Figure 3).

### 4.8 Enhanced canonical Wnt pathway in brain tissues of preeclamptic rats supplemented with omega‐3

**FIGURE 4:**
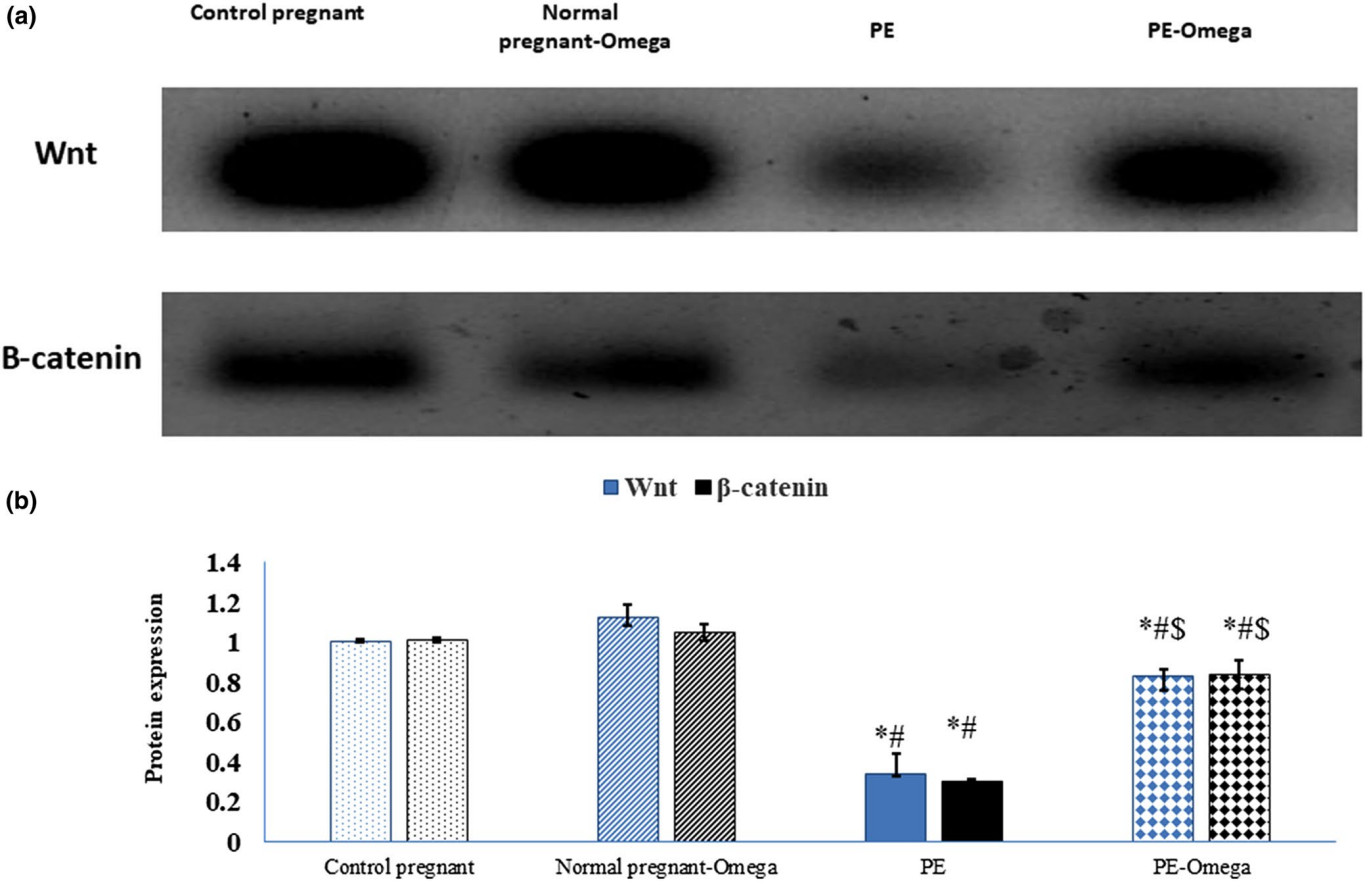
(a) Protein bands and (b) statistical analysis of Wnt and β‐catenin in relation to the house keeping gene β‐actin. Data are presented as mean ± SD (*n* = 6), *: statistically significant relative to the control group (*p* < 0.05), #: statistically significant relative to the normal pregnant‐omega group (*p* < 0.05), $: statistically significant relative to the PE group (*p* < 0.05)

Wnt functions by regulating the transcriptional coactivator β‐catenin, thus our results showed decreased expression of Wnt/β‐catenin in PE group compared to both normal control and normal pregnant rats supplied with omega‐3. In addition, the prenatal intake of omega‐3 in PE‐omega‐3 group resulted in increased protein level of Wnt and subsequent β‐catenin compared to PE group (Figure 4).

### 4.9 Omega‐3‐improved histopathological injury and cerebral microangiopathy in the brain tissue of preeclamptic rats

**FIGURE 5:**
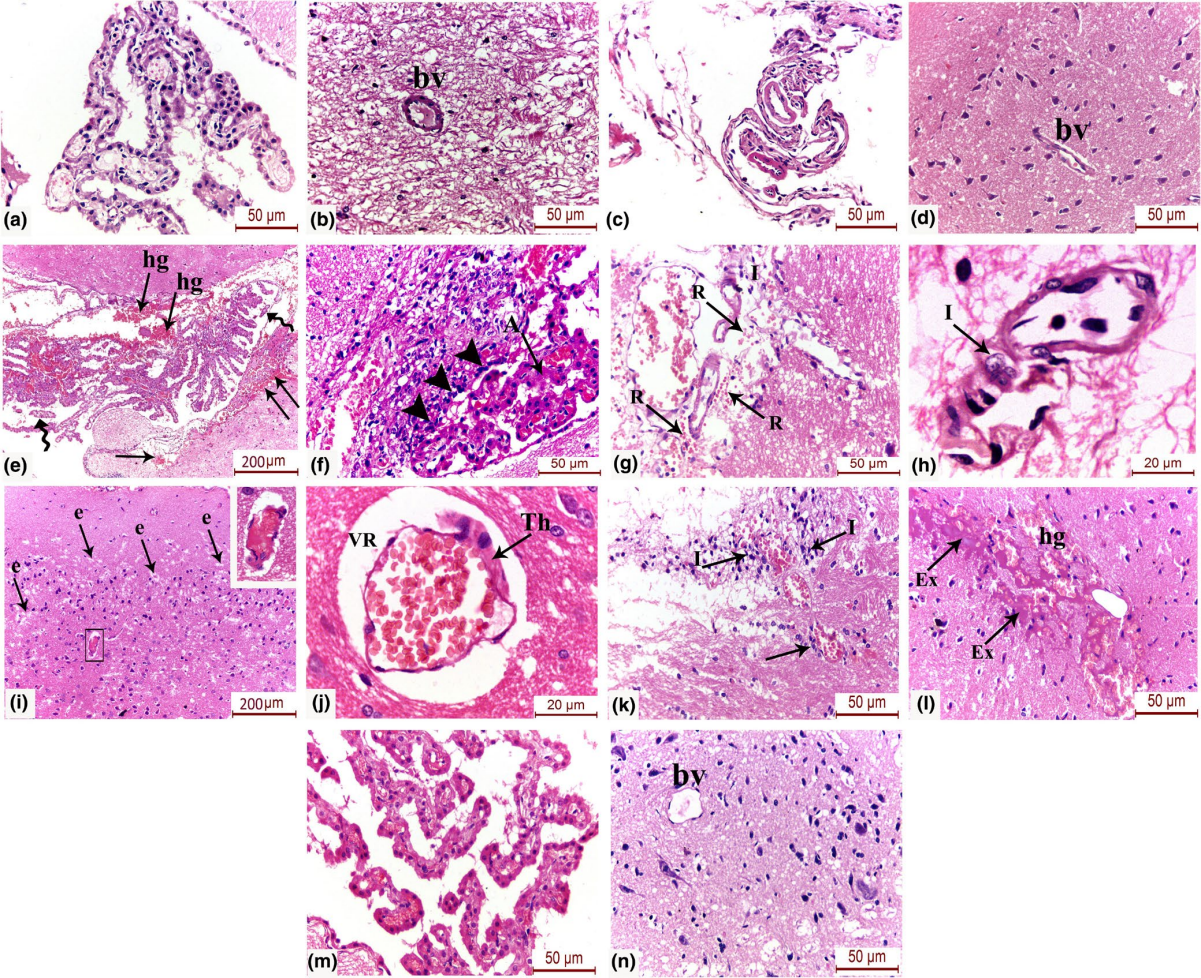
H&E‐stained sections of (a, b) choroid plexus and cortical blood vessels (bv) of the control group, (c, d) choroid plexus and cortical blood vessels (bv) of the omega‐3‐only group, (e–l) The preeclampsia group showing numerous pathological changes including (e) desquamated choroidal villi (spiral arrows) and areas of massive hemorrhage (hg) in the choroid plexus. The hemorrhage is also seen extending from the sub‐pial surface into the superficial cerebral cortex (double arrows), (f) High magnification demonstrates leucocyte adhesion (arrow heads) and choroid vascular angiopathy (A), (g) The pial blood vessels appear surrounded by red blood cell extravasation (R), petechial hemorrhages and scattered inflammatory cells (I), (h) High magnification demonstrates invasion of the pial capillary wall by inflammatory cells (I) with endothelial disfigurement (end), (i) The cerebral cortex shows areas of edema (e). The inset shows congested blood capillary with endothelial disruption along with widened perivascular space, (j) High magnification shows marked widening of the Virchow–Robin space (VR) with focal endothelial wall thickening (Th), (k) The cortical capillaries appear surrounded by mononuclear inflammatory infiltration (I), (l) The cerebral cortex shows hemorrhage (hg) and exudation (Ex), (m) The omega‐treated group shows improved histological structure of the choroid plexus vascular villi and ependymal cells with no obvious erythrocyte extravasation, (n) The cerebral blood vessels (bv) show healthy architecture

H&E‐stained sections were evaluated for assessment of histopathological changes in the cortical brain tissue, choroid plexus, and cerebral blood vessels of the different study groups. The preeclampsia group displayed substantial vascular angiopathy along with leucocyte adhesion, desquamation of choroid villi and areas of hemorrhage in the choroid plexus. The cortical blood vessels also showed endothelial disruption, widened perivascular (Virchow‐Robin) spaces and cerebral edema. Omega‐3 supplementation substantially ameliorated those histopathological changes with obvious improvement in the architecture of the brain tissue, choroid plexus vascular villi, ependymal cells, and cerebral cortical blood vessels (Figure 5a, b).

### 4.10 Increased immunohistochemical expression of claudin‐1 and claudin‐3 in the cerebral cortical capillaries and choroid villi as well as expression level of claudin‐5 in brain capillary fraction with omega‐3 supplementation

**FIGURE 6:**
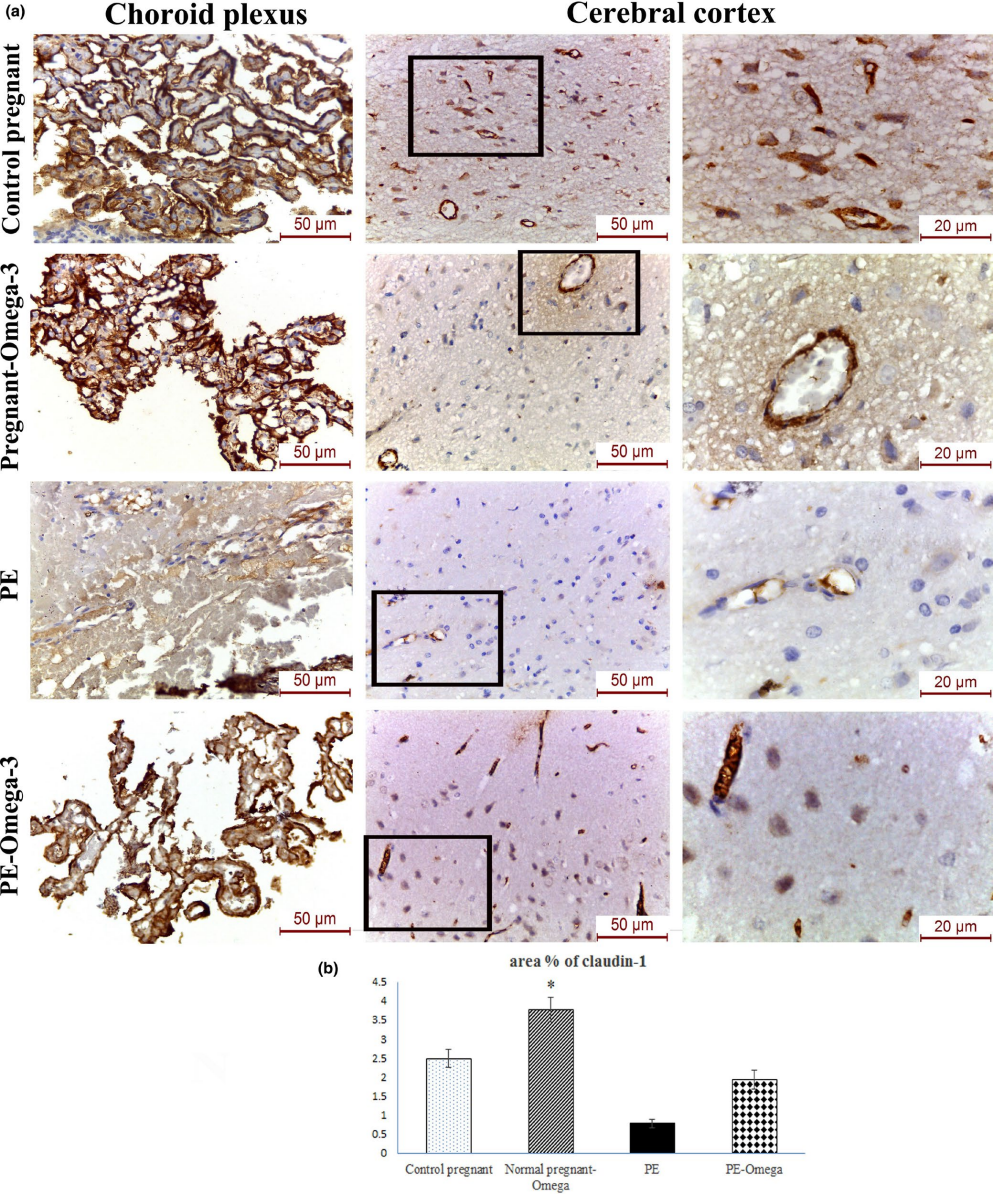
(a) Representative images of claudin‐1 immunohistochemical expression in the choroid plexus and cerebral vessels in the different study groups (scale bar 50 µm = magnification x400) (scale bar 20 µm = magnification x1000), (b) Area percent of claudin‐1 immunohistochemistry, Data are presented as mean ± SD (*n* = 6),*: statistically significant relative to the control group (*p* < 0.05), #: statistically significant relative to the normal pregnant‐omega group (*p* < 0.05), $: statistically significant relative to the PE group (*p* < 0.05) (arrows indicate positive immunoreactivity)

**FIGURE 7:**
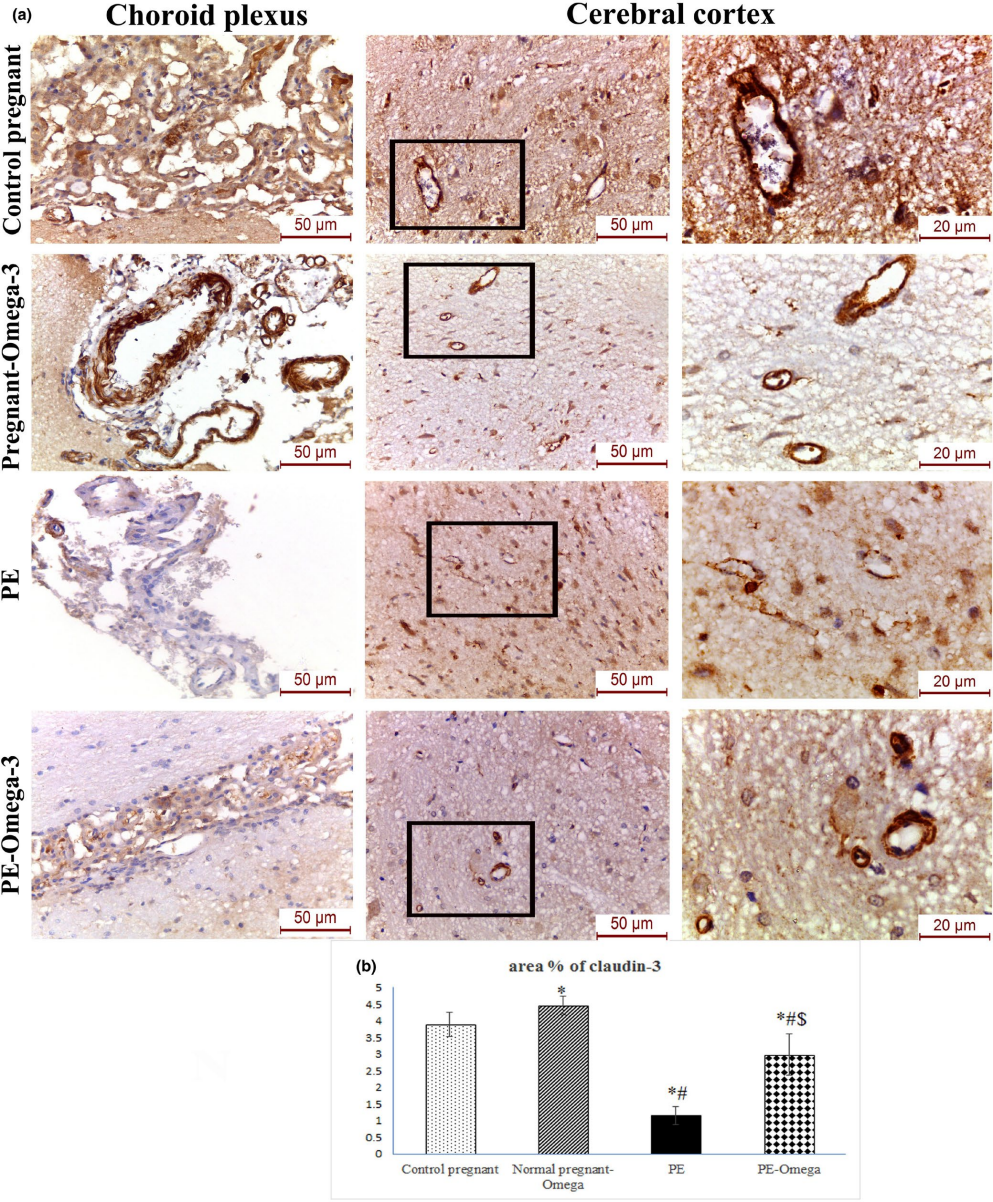
(a) Representative images of claudin‐3 immunohistochemical expression in the choroid plexus and cerebral vessels in the different study groups (scale bar 50 µm, magnification x400) (scale bar 20 µm = magnification x1000), (b) Area percent of claudin‐3 immunohistochemistry, Data are presented as mean ± SD (*n* = 6),*: statistically significant relative to the control group (*p* < 0.05), #: statistically significant relative to the normal pregnant‐omega group (*p* < 0.05), $: statistically significant relative to the PE group (*p* < 0.05) (arrows indicate positive immunoreactivity)

Owing to the importance of claudins as major constituents of the tight junctional complexes that regulate the permeability of epithelia and their roles as crucial transmembrane proteins in cell‐to‐cell adhesion, the current study has evaluated the changes in immunohistochemical expression of claudin‐1 and claudin‐3 in preeclampsia with and without supplementation of omega‐3. Our results have revealed statistically significant decrease in claudin‐1 and claudin‐3 immunohistochemical expression of the PE group compared to the control pregnant and normal pregnant‐omega groups. Prenatal omega‐3 supplementation resulted in significant increase in claudin‐1 (Figure 6a,b) and claudin‐3 (Figure 7a,b) immunohistochemical expression in both the cerebral cortical capillaries and choroid villi which essentially denote parallel improvement in BBB function.

Expression levels of claudin‐5 was significantly decreased in the PE group (0.36 ± 0.06) compared to the control pregnant (1.01 ± 0.02) and normal pregnant‐omega groups (1.04 ± 0.03). However, our results revealed statistically significant increase in claudin‐5 expression of the PE‐omega (0.84 ± 0.06, *p* < 0.001) compared to the PE groups.

### 4.11 Effect of omega‐3 on cerebral angiogenesis in the brain tissues of preeclamptic rats

**FIGURE 8:**
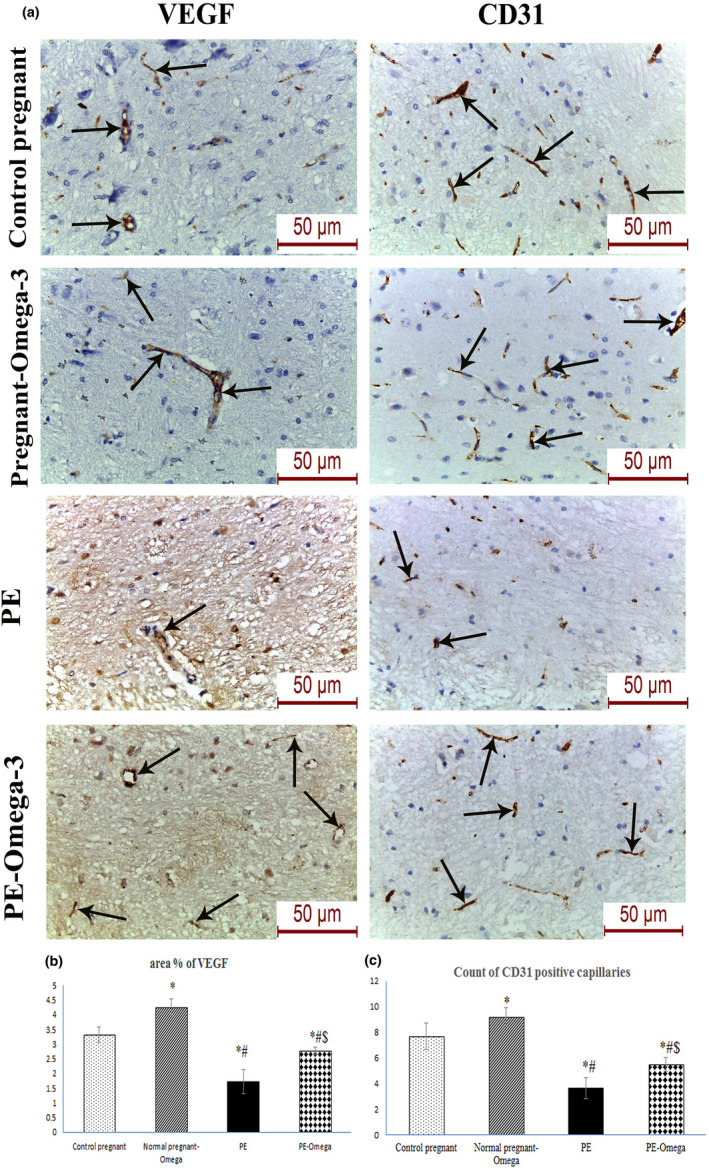
(a) Representative images of VEGF and CD31 immunohistochemistry in the different study groups (scale bar 50 µm = magnification x400), (b) Area percent of VEGF immunohistochemical expression, (c) Count of CD31 positive capillaries (*n* = 6), Data are presented as mean ± SD (*n* = 6),*: statistically significant relative to the control group (*p* < 0.05), #: statistically significant relative to the normal pregnant‐omega group (*p* < 0.05), $: statistically significant relative to the PE group (*p* < 0.05) (arrows indicate positive immunoreactivity)

Evaluation of the angiogenic factors VEGF and CD31 revealed statistically significant increase in the count of CD31 positive capillaries and area percent of VEGF expression in the omega‐3‐treated group relative to the PE group, which indicates improved microvascular and capillary angiogenesis (Figure 8).

### 4.12 Correlation

**FIGURE 9:**
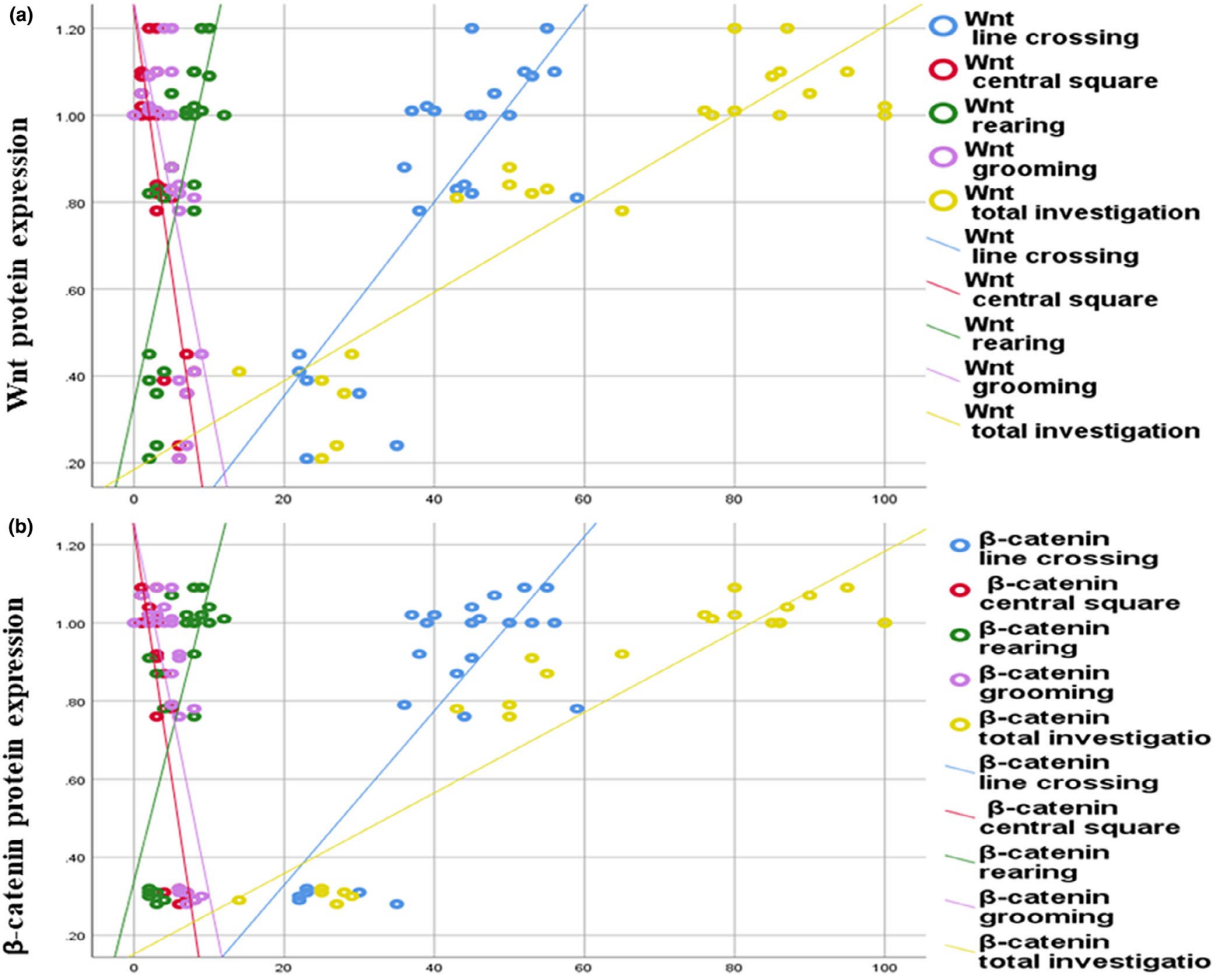
(a) Correlation study between protein expression level of Wnt and cognitive functions, Wnt displayed strong positive correlation with No of line crossing (*p *= 0.000), No of rearing (*p *= 0.000), percentage of total investigation time (*p *= 0.000), negative correlation with time in central square (*p *= 0.000), and No of grooming (*p *= 0.000). (b) Correlation study between protein expression level of β‐catenin and cognitive functions. β‐Catenin displayed positive correlation with no of line crossing (*p* = 0.000), no of rearing (*p* = 0.000), and % of total investigation time (*p* = 0.000), negative correlation with time in central square (*p* = 0.000), and no of grooming (*p* = 0.000)

We further determined the correlation between protein expression level of Wnt and β‐catenin from one side and cognitive functions on the other side. The perturbation of Wnt/β‐catenin signaling in brain tissues was correlated with changes observed in cognitive functions. Wnt displayed strong positive correlation with No of line crossing (*r* = 0.798; *p *= 0.000), No of rearing (*r* = 0.780; *p *= 0.000) and % of total investigation time (*r* = 0.909; *p *= 0.000) and negative correlation with time in central square (*r* = −0.832; *p *= 0.000), and No of grooming (*r* = −0.676; *p *= 0.000) (Figure 9a).

**FIGURE 10:**
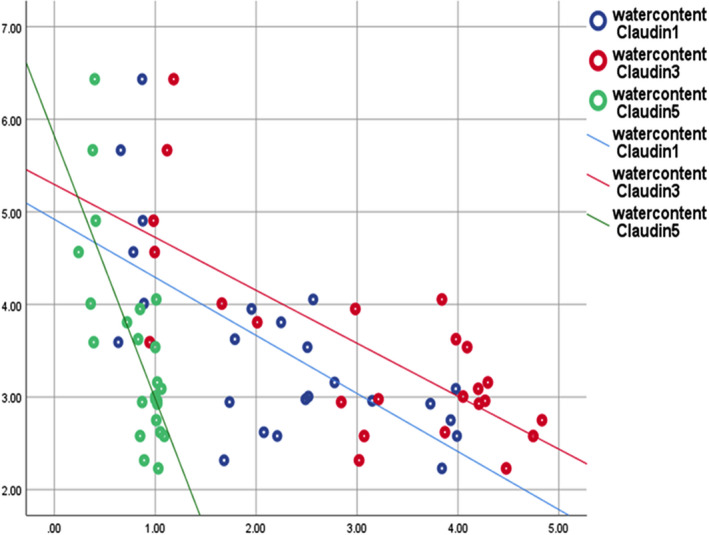
Strong negative correlation between water content and area % of claudin‐1,‐3, and relative expression of claudin‐5 (*r* = −0.672; *p* = 0.000, *r* = −0.733; *p* = 0.000, and *r* = −0.762; *p* = 0.000, respectively)

In addition, β‐catenin displayed positive correlation with No of line crossing (*r* = 0.803; *p *= 0.000), No of rearing (*r* = 0.745; *p *= 0.000) and % of total investigation time (*r* = 0.925; *p *= 0.000) and negative correlation with time in central square (*r* = −0.879; *p *= 0.000), and no of grooming (*r* = −0.720; *p *= 0.000) (Figure 9b), whereas, water content showed negative correlation with area percent of claudin‐1, −3, and −5 (*r* = −0.672; *p *= 0.000, *r* = −0.733; *p *= 0.000 and *r* = −0.762; *p *= 0.000, respectively) (Figure 10).

## 5 DISCUSSION

L‐NAME‐induced hypertension in pregnant rats, mimics preeclampsia‐mediated vasculopathy, diminishes placental blood flow, and causes placental hypoxia (Chaiworapongsa et al., 2014). One of the substances secreted by placenta secondary to hypoxia is sVEGFR‐1 (Ahmad & Ahmed., 2004). Soluble VEGFR‐1 is also known as soluble fms‐like tyrosine kinase (sFlt1‐14 or sFlt1‐e15a) (Failla et al., 2018), it could bind to and antagonize the activity of all isoforms of VEGF and placenta growth factor (Kendall et al., 1996). VEGF is a central requirement for stabilization of endothelial cells in mature blood vessels and it is particularly important in the health of the fenestrated and sinusoidal endothelium found in the renal glomerulus, brain, and liver organs (Maynard & Karumanchi, 2011). VEGF blockade represents a crucial factor in the pathophysiology of preeclampsia (Younes & Ryan, 2019). Current data showed significant decrease in area % of VEGF immune‐staining in PE group compared to both control normal and normal pregnant rats supplemented with omega‐3.

High level of sFlt1/sVEGFR‐1 found in preeclampsia is involved in vascular dysfunction, modulation of endothelial cell migration and sprouting (Failla et al., 2018). The high serum concentration of TNF‐α is also akin to the development of preeclampsia (Brogin Moreli et al., 2012). Ahmad and Ahmed (2004) reported enhanced release of sVEGFR‐1 from normal placental explants secondary to TNF‐α. Even more, placental hypoxia increases production of TNF‐α (Weel et al., 2016), both act in synergistic way potentiating the release of sVEGFR‐1(Ahmad & Ahmed, 2004).

Increased circulating sFlt1 in preeclampsia is implicated in development of vasculopathy that underline proteinuria, disruption of the BBB and cerebral edema (Miller., 2019). Moreover, sFlt1 infusion to pregnant rats induces preeclampsia‐like syndrome (Wallace et al., 2018). The results of the present work showed increased sFlt1 and TNF‐α in the PE group compared to control pregnant group.

The low level of DHA may be one of the reasons that induce sVEGFR‐1 release into circulation (Kulkarni et al., 2011) as a result of endothelial dysfunction accompanying pregnancy‐induced hypertension (Burchakov et al., 2017). Moreover, DHA stimulate VEGF mRNA expression and protein secretion and decrease level of sVEGFR‐1 which is the antagonist of VEGF (Johnsen et al., 2011). Concordant with our results, previous studies have also illustrated that decreased VEGF together with increased expression of sFlt‐1 are the cardinal factors of endothelial dysfunction in preeclampsia. There is additional experimental evidence that the interference with VEGF signaling could mediate endothelial dysfunction in preeclampsia (Maynard & Karumanchi, 2011) and that the cognitive alterations present in children born from preeclamptic pregnancies are attributed to impaired cerebral VEGF and angiogenesis (Lara et al., 2018).

Those results pave the way for novel potential preeclampsia therapies directed at restoring normal angiogenic balance in the maternal circulation akin to the previous lines demonstrating employment of VEGF agonists for ameliorating hypertension and proteinuria in murine models of preeclampsia (Bergmann et al., 2010; Li et al., 2007). Other strategies include the use of monoclonal antibodies to sFlt1 or small‐molecule inhibitors of sFlt1, or agents that enhance endogenous VEGF production. Such therapies can dramatically transform the management approach of preeclampsia (Karumanchi, 2016, 2018).

In the present study, estimated lipid profile levels (cholesterol, LDL, and triglyceride levels) in L‐NAME injected pregnant female rats showed significant increase while the protective HDL was significantly decreased compared to control pregnant rats. Aluko et al., (2018) reported that nitric oxide acts as a principal anti‐atherosclerotic factor and inhibition of NO synthase‐enhanced atherosclerosis in experimental animals. NO has beneficial effects on lipid metabolism through its ability to activate hepatic sterol regulatory element‐binding protein 2; the transcriptional factor regulating cholesterol metabolism and expression of LDL receptors (Luo et al., 2020). The latter enhances the uptake of cholesterol by hepatic cells, thus it maintains normal blood cholesterol level (Aluko et al., 2018).

CD31 is a key endothelial adhesion protein mediating endothelial integrity and regulation of bioavailability of NO, and angiogenesis (Bagi et al., 2005; Liu et al., 2006; Maas et al., 2005). The histopathological alterations of choroid plexus and cerebral microvasculature in the preeclampsia group of the current study reflect considerable BBB dysfunction which essentially leads to vasogenic edema and subsequent hypertensive encephalopathy.

Consistent with our results, previous studies have demonstrated that preeclampsia causes loss of the endothelial marker CD31 in the brain capillaries (Hecht et al., 2017). Studies of CD31 knockout mice have also revealed marked enhancement of vascular permeability (Carrithers et al., 2005; Maas et al., 2005; Wong et al., 2005).

Hypertension compromises the ability of blood flow to match neuronal metabolic demand and lead to chronic hypoperfusion that may predispose to white matter lesions and decline in cognitive function (Cipolla et al., 2018). In this context, the results of cognitive function tests in the present study yielded a significant affection in PE group compared to control pregnant rats. In addition, EEG amplitude was significantly increased in PE rat compared to control pregnant rats. In another study conducted in PE rats, the recorded EEG was the highest in amplitude, correlated with the severity of PE and related to exaggerated systemic pro‐inflammatory state evident by high levels of TNF‐α (liu et al., 2016). In addition, preeclampsia may induce seizures by developing abnormal hyper‐synchronous electrical activity of neuronal networks in the brain tissues (Lonsdale et al., 2007). Currently, the recorded EEG waves were the highest in amplitude in PE group compared to control pregnant.

In neonatal and adult models of cerebral ischemia, degradation of TJs and basal lamina proteins causes disturbed integrity of the BBB (Zhang et al., 2020). MMP‐9 contributes to the early BBB opening, leakage, and brain edema that will result in abnormal neurological function (Zhang et al., 2016). Meanwhile, using a broad‐spectrum MMP inhibitor reduced brain edema and restored the integrity of BBB by enhancing expression tight junction proteins and promoting angiogenesis (Yang et al., 2013).

Thus, identifying therapies that will preserve BBB integrity becomes a new concern. Treatment with omega‐3 reduced the infarct area and mitigated BBB disruption in adult rats (Hong et al., 2015) and in neonatal brain injury induced cerebral hypoxic‐ischemia (Zhang et al., 2016). Herein, omega‐3 supplementation decreased the protein level of MMP‐9 in brain tissues of adult pregnant female rats exposed to preeclampsia. The decreased wet brain weight, water contents and enhanced expression of tight junction proteins in PE‐omega‐3 was also observed compared to PE group.

Omega‐3 supplementation exerts antioxidant property. DHA, a component of omega‐3, incorporates to cerebral vascular endothelial cells from the systemic circulation, enhances membrane fluidity and the function of BBB (Hong et al., 2015). Aside from the antioxidant effects, omega‐3 fatty acids could decrease lipid profile that was also confirmed by the current work. In addition, omega‐3 uniformly inhibits cytokine production (lnahdi et al., 2019).

Wnt activation leads to a stabilization of β‐catenin, and Wnt/β‐catenin signaling pathway is implicated in expression of capillary tight junction proteins in the developing brain (Tran, Zhang, et al., 2016). Meanwhile, in adult life, the dysregulation of Wnt/β‐catenin is involved in BBB breakdown (Laksitorini et al., 2019).

Khiem and colleagues (2016) demonstrated for the first time the role of β‐catenin signaling in maintenance of BBB integrity in adult mice. Increased β‐catenin levels will translocate from the cytosol to the nucleus where it acts to modulate transcription of target genes that regulate the expression of tight junction proteins such as claudin‐1 and 3 (Alvarez et al., 2011). Khiem study (2016), concluded that claudin‐1 is a transcriptional target of β‐catenin signaling in brain endothelial cells. Claudin‐5, in specific, could maintain microvasculature integrity of the brain TJs and is crucial for their correct organization. Moreover, claudin‐5 has been linked to Alzheimer's disease and edema (Ma et al., 2017). Currently, we also found changes in the expression of claudin‐5 were negatively correlated with brain water content.

Omega‐3 intake could significantly restore expression of the components of the tight junction proteins (Zhang. et al., 2020), and DHA inhibits the caveola‐mediated transcytosis to maintain BBB integrity (Wang et al., 2020). Currently, the PE‐treated group with omega‐3 showed enhanced expression of Wnt/β‐catenin that was positively associated with increased area percent of claudin‐1 and −3 in brain tissues as well as relative expression of claudin‐5.

The present study reported that regular daily supplementation of omega‐3 fatty acids was able to decrease the mean systolic blood pressure of PE‐omega‐3 group compared to PE rats, which was also previously reported by Watanabe and colleagues (2017). In the present study, short‐term intake of omega‐3 started before induction of PE was able to enhance expression of eNOS and downregulate iNOS in PE‐omega‐3 compared to PE rats. In agreement with Mori et al., (2018), omega‐3 supplementation in adult rats was associated with reductions of iNOS expression, as the incorporation of these fatty acids in neurons and different glial cells may lead to potent antioxidant, anti‐inflammatory effects. Adding to that, omega‐3 intake could upregulate the canonical Wnt signaling in PE and subsequent increase tight junction proteins in choroid plexus and cerebral microvasculature.

## 6 CONCLUSION

Disruption of tight junction structure in BBB leads to brain edema and enhances vascular leakiness that was associated with downregulation of Wnt signaling and decreased its downstream claudin‐1, ‐3, and ‐5. Thus, regulating Wnt signaling pathways is of interest therapeutically to maintain integrity of BBB. In this work, omega‐3 supplementation limited the inflammation and oxidative damage with subsequent decreased iNOS and increased eNOS levels in brain tissues of preeclamptic rats. In addition, it enhanced Wnt/β‐catenin signaling pathway.

## CONFLICT OF INTEREST

The authors declare no conflict of interest.

## AUTHOR CONTRIBUTION

Conceptualization and design of the study: M. Gamal and A.M. ShamsEldeen. All authors contributed to acquisition of data, analysis, interpretation of data, and approved the final version for submission. Drafting and Revision of the article: M. Gamal, B.E Aboulhoda, and A.M. ShamsEldeen.
